# Right atrial myxoma with glandular differentiation: A rare entity in pediatric age group

**DOI:** 10.4103/0974-2069.74046

**Published:** 2010

**Authors:** Saumya R Mallick, Prasenjit Das, Bhaskar Shukla, SS Kothari, V Devagourou, Ruma Ray

**Affiliations:** Department of Pathology, All India Institute of Medical Sciences, New Delhi, India; 1 Department of Cardiovascular Surgery, All India Institute of Medical Sciences, New Delhi, India

**Keywords:** Cardiac myxoma, lepidic cells, divergent, glandular differentiation, children

## Abstract

Cardiac myxomas (CMs) account for nearly half of the primary cardiac tumors in the elderly. They arise from sub-endocardial “reserve” or lepidic” cells, which may show divergent differentiation. We describe a CM with glandular differentiation in the right atrium of a 10-year-old child who presented with respiratory distress on exertion, of 2 months duration. On echocardiography, two large interconnected masses measuring 34×30 mm and 20×17 mm were seen to arise from the free wall of the right atrium. Cut surface of the excised mass was myxoid with areas of calcification. On microscopy, there were typical features of a myxoma with prominent glandular differentiation and characteristic immunophenotype. The case is being reported due to its rarity in pediatric age group as well as its glandular differentiation, which must be recognized as a spectrum of histomorphologic diversity and must not be mistaken for a metastatic adenocarcinoma.

## INTRODUCTION

Primary tumors of the heart are rare, with a reported incidence in the range of 0.0017-0.33% in autopsy series, amongst which cardiac myxoma (CM) is the commonest. It commonly occurs in females during third to fifth decade and is rare in pediatric population, in whom it has been reported to occur only up to 7%. Most (75%) of the myxomas arise from the left atrium near the fossa ovalis of inter-arterial septum, while the others arise within the right atrium. Majority of them present sporadically; however, they can present with Carney’s complex.[1] In general, myxomas are mesenchymal in origin. Glandular differentiation in a myxoma is rare and has been occasionally reported in association with Carney’s complex.[2–4] We present a case of myxoma showing glandular differentiation arising in the right atrium in a child, with a detailed histochemical and immunohistochemical analysis.

## CASE REPORT

A 10-year-old boy presented with gradually progressing respiratory distress on exertion, of 2 months duration. A two-dimensional echocardiography showed two large interconnected masses measuring 34×30 mm and 20×17 mm, attached to the free wall of right atrium close to inferior vena cava. The lesion was surgically resected and sent for histopathologic examination. There was no similar or related diseases noted in the family.

### Pathologic findings

**Figure 1:**
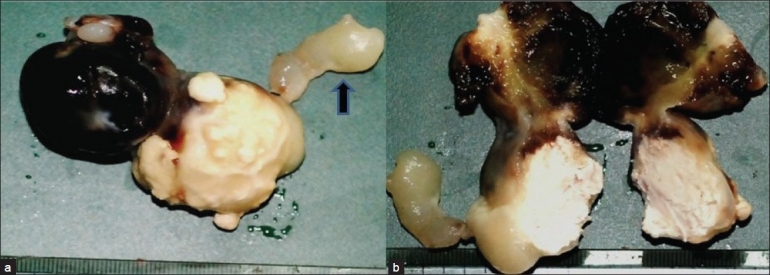
Gross photomicrograph shows a dumbell shaped gelatinous mass with attached short pedicle (arrow). Large areas of hemorrhage are also identified (a and b)

Grossly, the tumor comprised two well–circumscribed unencapsulated globular soft tissue masses which were interconnected by a short pedicle. The tumor was 60×40×30 mm in dimension. Cut surface of the tumor was gray-white and had gelatinous and calcified areas [Figure 1].

**Figure 2:**
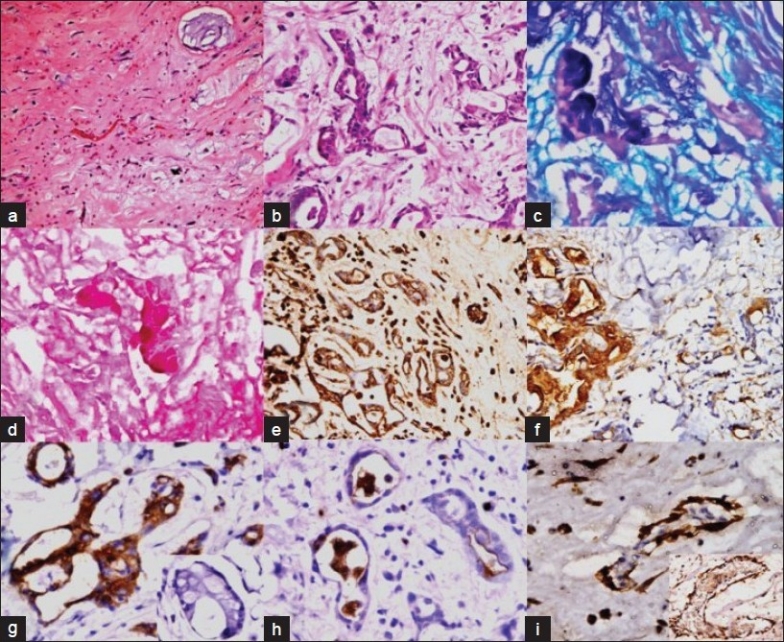
Photomicrograph shows a classical CM (a; ×40). Glandular structures can be seen within the myxoma surrounded by stellate cells and myxoid areas (b; H and E, ×200). Special stains show mixed intra and extracellular acid mucin (c; AB-PAS, ×200) as well as neutral mucin (d; mucicarmine ×200). The glandular structures and stellate cells show positivity for vimentin (e; ×40), EMA (f; ×40), CK-7 (inset: CK-20 stain is negative) (g; ×400; inset: ×400) and CEA (luminal positivity) (h; ×400). Calretinin positivity is seen in epithelial cell nuclei (inset: calretinin positivity in vessel wall) (i; ×200; inset: ×200)

Hematoxylin and eosin stained slides (H and E) showed a hypocellular tumor comprising stellate or lepidic cells in a myxomatous background, showing evidence of old hemorrhage and minimal fibrosis. These cells had scant to moderate amount of eosinophilic cytoplasm and elongated to star shaped nuclei. A few thin– as well as thick–walled blood vessels were also identified. The blood vessels in some areas showed subendothelial myointimal proliferation, surrounded by mucin lakes. Glandular structures were lined by cuboidal to tall columnar cells interspersed with mucin droplets and thin basophilic secretions in the lumen. Some of these glands were seen floating inside a pool of mucin. Focal conglomeration and back to back arrangement of the glands with formation of cribriform structures were noted. The lepidic cells were found evenly distributed, whereas the glandular component was confined to the pedicle of the tumor [Figure 2a and b].

### Histochemical staining

Alcian blue-periodic acid Schiff (AB-PAS) and mucicarmine staining was performed in the representative sections. There were large pools of extracellular mucin positive for both PAS and AB stains at pH 2.5, indicating its acidic nature. The glandular epithelium showed basal positivity for PAS and apical positivity for AB. Mucicarmine stain positivity correlated with the pattern of positivity of AB [Figure 2c and d] [Table 1].

**Table 1:** Details of histochemical staining pattern of the tumor cells

Stains	Gland	Stroma	Vessel
AB-PAS	Goblet cells- AB positive	Acid mucin	Subintimal
	Mucin secreting cells –	positive	region positive
	PAS positive		
Mucicarmine	Glands focal positive	Focal positive	Negative

### Immunohistochemistry

Immunohistochemical stains for pan-cytokeratin (CK) (Dako, Denmark; 1:100), CK-7 (Dako, Denmark; 1:100), CK-20 (BioGenex, CA, USA; 1:50), carcino-embryonic antigen (CEA) (BioGenex, CA, USA; 1:100), vimentin (DBS, CA, USA; 1:100), S-100 protein (Dako, Denmark; 1:800), calretinin (BIO SB, CA, USA;1:100), desmin (Biocare, CA, USA; 1:50) and epithelial membrane antigen (EMA) (Dako, Denmark; 1:100) were performed on representative sections after antigen retrieval.

Vimentin, EMA, pan-CK, CK-7, CEA and calretinin were positive in the glandular structures and variably in stellate cells and blood vessels [Figure 2e–i]. Desmin was focally positive in the stellate cells and blood vessels [Table 2].

**Table 2:** Details of immunohistochemical stains of tumor components

Immunostain	Glands	Stroma	Blood vessels
CEA	Apical surface	Negative	Negative
	cytoplasm		
EMA	Cell surface	Focally stellate	Negative
	cytoplasm	cells positive	
Vimentin	Cytoplasmic	Stellate cells	Positive
	positivity	positive	
S–100	Focally glands	Occasional stellate	Negative
	positive	cells positive	
Calretinin	Positive	Stellate cells	Outer wall cells
		positive	positive
CK	Positive	Negative	Negative
CK-7	Strongly positive	Negative	Negative
CK-20	Negative	Negative	Negative

## DISCUSSION

Though first described in 1845, epithelial differentiation in a myxoma was reported after a long period in 1946 by Anderson *et al*.[1] Though myxomas have been reported in children aged as early as 1 month,[5] to the best of our knowledge, a right atrial myxoma with glandular differentiation has not been reported in children and the index case represents the first documentation of the same.

Atrial myxoma in younger patients is commonly associated with familial syndromes as Carney’s complex with frequent tumor recurrence, more gelatinous appearance and little fibrosis.[1] In the index case, the other features of a familial link such as spotty pigmentation, endocrine abnormalities or myxoid neurofibromas were not identified. On histologic examination, abundant extracellular and peri-vascular acidic mucin was identified with minimal fibrosis, indicating the probability that fibrosis in a myxoma is related to the chronicity of the disease.

There are many hypotheses behind the glandular differentiation in a myxoma:

1) Divergent differentiation of sub-endocardial lepidic cells, 2) the possibility of entrapped foregut rests or 3) progressive differentiation of myxoma into glands. Initially, myxoma was also considered as an organizing thrombus rather than a tumor; however, now it has been accepted as a neoplasm.[2] The histochemical and immunohistochemical profile of this tumor, as seen in the index case or of those reported in literature, shows that there is positivity for both mesenchymal and epithelial differentiation, indicating that the primitive reserve cells are the possible source.[2] In the index case, pan CK and CK–7 were strongly positive along with EMA and CEA, whereas CK–20 was negative in the glandular structures, indicating a possibility of entrapped foregut rest origin of this tumor. Such a link was also identified by Pucci *et al*.[6] The tumor components also showed clear positivity for calretinin, while in the above mentioned report the authors failed to demonstrate a mesothelial link.[6] In a mesothelioma, all the above mentioned markers can be positive; however, they are rarely positive for mucicarmine. Hence, stating that myxomas arise from mesothelial rests, is also not out of controversy.[3] It can further be argued if myxomas are a part of teratoma or not; absence of squamous islands or any other mesenchymal elements rule out this possibility. In a report by Linder *et al*., the authors described such myxomas as “congenital endodermal heterotopia of the AV node,” based on the positivity for PAS-D and CEA;[7] however, this hypothesis does not hold true due to absence of any other element. Hence, though it is apparent that these glandular structures are footprints of the divergent differentiation of reserve cells, from the histochemical and immunohistochemical pattern we cannot prove or disprove any of these hypotheses.

In the index case the glands were either lined by tall columnar or cuboidal cells with focal nucleomegaly and stratification, without a clear evidence of cytological atypia. Atypical mitosis was not identified. Though an embolus from this tumor in cerebral vessels can show proliferative activity[1] or very rarely an infiltrating adenocarcinoma had been reported inside a myxoma,[6] absence of cytological atypia or invasive glandular structures inside the wall of adjacent cardiac chamber in the present case rules out these possibilities. However, unless recognized as part of the tumor, these glands may lead to an erroneous diagnosis of secondary adenocarcinoma.[4]

Pucci *et al*., in their case series, elegantly tabulated around 28 reports of atrial myxomas with glandular differentiation in adults.[6] However, none of these reported cases was noted in a child.

## CONCLUSIONS

Glandular differentiation in a CM, though rare, is a possible morphologic diversity arising out of intracardiac endodermal heterotopia or represents entrapped foregut rests. This morphologic diversity is extremely rare in pediatric age group. It is important to recognize this entity, as failure to do so may lead to an erroneous diagnosis of metastasis from a mucin secreting adenocarcinoma in the atrium.
